# Argyrophilic grain disease is common in older adults and may be a risk factor for suicide: a study of Japanese forensic autopsy cases

**DOI:** 10.1186/s40035-023-00352-2

**Published:** 2023-04-01

**Authors:** Koji Yoshida, Yukiko Hata, Shojiro Ichimata, Keitaro Okada, Naoki Nishida

**Affiliations:** 1 Department of Legal Medicine, Faculty of Medicine, University of Toyama, 2630 Sugitani, Toyama-shi, Toyama, 930-0194 Japan; 2 Tanz Centre for Research in Neurodegenerative Disease, Krembil Discovery Tower, University of Toronto, 60 Leonard Ave Toronto On, Toronto, ON M5T 0S8 Canada; 3 Department of Laboratory Medicine and Pathobiology, University of Toronto, Toronto, ON Canada

**Keywords:** Amygdala, Argyrophilic grains, Dementia, Forensic autopsy, Psychiatric disorder, Suicide

## Abstract

**Background:**

Neuropathological diagnosis of argyrophilic grain disease (AGD) is currently based primarily on the combination of argyrophilic grain (AG) visualized using Gallyas–Braak silver staining, phosphorylated tau-positive pretangles, coiled bodies, and ballooned neuron detection. Although AGD is common in patients with dementia and/or prominent psychiatric symptoms, whether it is a distinct neurological disease entity or a by-product of the aging process remains unclear.

**Methods:**

In 1449 serial forensic autopsy cases > 40 years old (823 males and 525 females, aged 40–101 years, mean age 70.0 ± 14.1 years), we examined the frequency and comorbid pathology of AGD cases and investigated the clinical appearance by comparing those with non-AGD cases using the propensity score.

**Results:**

Of the 1449 cases, we detected 342 AGD cases (23.6%; mean age 79.7 years; 177 males and 165 females). The AGD frequency and stage increased with age (*P* < 0.001). Among AGD cases, 80 (23.4%) patients had dementia, and 51 (15.2%) had a history of psychiatric hospital visits. The frequency of suicide and history of psychiatric disorders were significantly higher in AGD cases than in AGD-negative cases, matched for age, sex, and comorbidity pathology, with a relative risk of suicide of 1.72 (1.30–2.26). The frequency of suicide was significantly higher in AGD cases than in non-AGD cases in female but not male cases. The relative risk of suicide increased to 2.27 (1.20–4.30) and 6.50 (1.58–26.76) in AGD patients with Lewy and progressive supranuclear palsy pathology, respectively, and decreased to 0.88 (0.38–2.10) in those with advanced AD pathology. In AGD cases, 23.4% had dementia; however, the difference was not significant after controlling for age, sex, and comorbid pathology.

**Conclusion:**

Our study demonstrated that AGD is a significant and isolated risk factor for psychiatric hospital visits and suicide completion. In older adults, AGs may contribute to the progression of functional impairment of the limbic system, which leads to psychiatric disorders and suicide attempts.

**Supplementary Information:**

The online version contains supplementary material available at 10.1186/s40035-023-00352-2.

## Introduction

**Fig. 1:**
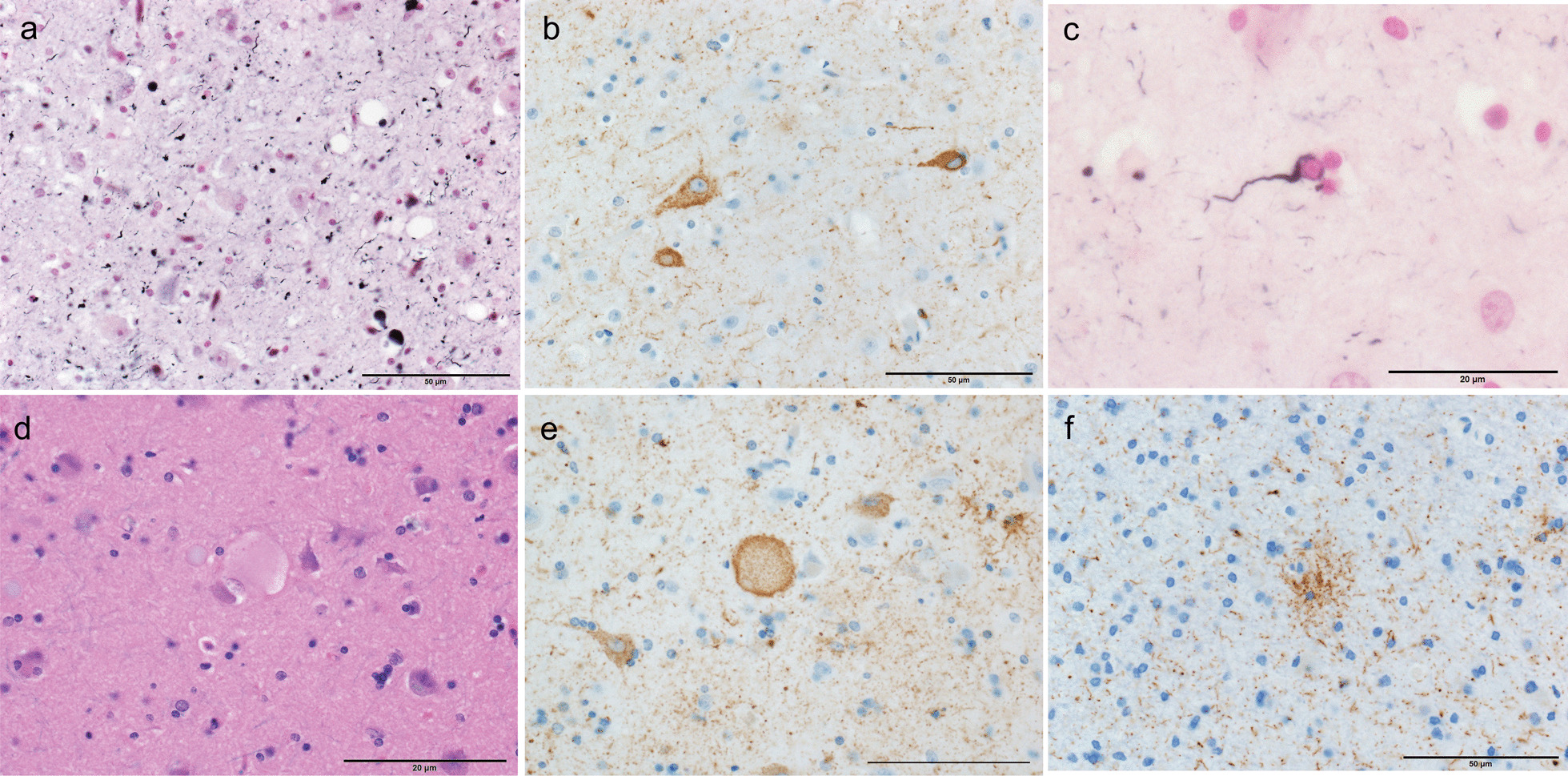
Pathological appearance of argyrophilic grain disease. Photomicrograph of Gallyas–Braak (**a**, **c**), AT8 (**b**, **e**, **f**), and luxol fast blue-hematoxylin and eosin (**d**). **a** Argyrophilic grains. **b** AT8-positive pretangle in the amygdaloid body. **c** Oligodendroglial coiled body. **d**, **e** Achromasic ballooned neuron. **f** Granular astrocyte. Scale bars, 50 µm (**a**, **b**, **f**) and 20 µm (**c**–**e**)

Argyrophilic grains (AGs) were first reported by Braak and Braak in 1987. Their pathological features are punctate or filiform structures in the neuropil, which can be visualized using Gallyas–Braak silver staining [1]. Previous studies have shown a high frequency of AGs among older adult patients with dementia [2, 3], which has led to AGs or argyrophilic grain disease (AGD) being proposed as an isolated neuropathological entity [2–5]. Neuropathological diagnosis of AGD is currently based on a specific pathological appearance: AGs consisting of four-repeat isoform tau protein [6], neuronal cytoplasmic tau-positive inclusions referred to as “pretangles”, and oligodendroglial coiled bodies [7]. Tau-positive granular/fuzzy astrocytes [8], which were initially reported by Botez et al. [9] as bush-like astrocytes, as well as ballooned neurons, are also considered as associated lesions of AGD [8] (Fig. 1). However, granular/fuzzy astrocytes are now defined as age-related tau astrogliopathy [10] and thus, there is currently no AGD-specific tau-positive astrocytic lesion.

Earlier studies have established that AGs are commonly distributed in various areas of the limbic system [1–5]. Saito et al. proposed the staging paradigm of AGs: AGs start with the involvement of the ambient gyrus and amygdala (stage 1: ambient stage), then extend into the posterior parahippocampal gyrus and medial anterior temporal pole (stage 2: medial temporal stage), and finally extend to the basal forebrain and anterior cingulate gyrus (stage 3: frontal stage) [11]. Although AGD has four-repeat tauopathy, similar to progressive supranuclear palsy (PSP) and corticobasal degeneration (CBD), AGs are rarely found in the basal ganglia, brain stem nuclei, or cerebellum [12]. However, rare cases with an extensive distribution of AGs in the neocortex have been reported [13].

A clinical definition of AGD has not yet been established. Although AGD is considered a distinct clinicopathological entity and a cause of dementia [14], this is likely because of the heterogeneous clinical presentation and the frequent overlap with other neurodegenerative diseases [8, 15]. The relatively high frequency of AGD in cognitively normal older adults over 60 years old prompts the question of whether AGD is a distinct neurological disease entity or a by-product of the aging process [16–18]. AGD has been reported in subjects with a relatively late onset of prominent psychiatric symptoms, which may be associated with the anatomical distribution of AGs [19–21]. Although neuropathological investigations targeting neuropsychiatric disorders, including suicide, and neurodegenerative diseases are scarce, they may be valuable [8, 22–24]. In our previous investigation in younger subjects aged under 40 years, Alzheimer’s disease (AD)-related pathology was not shown to be a significant accelerating factor for suicide [25], and we previously observed AGD in a suicide victim aged 40 years [26]. In the present study, we investigated a large number of serial forensic autopsy cases to reveal the frequency and comorbid pathology of AGD and examine the clinical appearance by comparing AGD cases with non-AGD cases.

## Materials and methods

### Subjects

We reviewed the archives of all 1982 medicolegal autopsy cases in our department between 2007 and 2020. Of these cases, brain specimens from 1449 people aged over 40 years (881 males and 568 females, aged 40–101 years, mean age 70.0 ± 14.1 years) without severe injury or severe postmortem degeneration (e.g., liquefaction preventing histological analysis), large chronic infarcts (> 1 cm), and/or multiple lacunas over three were examined. A total of 359 cases died of natural causes, and 669 cases had an accidental traumatic death, such as a fall, traffic accident, burning, drowning, and hypothermia. Suicide accounted for 395 cases, and 23 cases died by homicide. There were 1103 cases (625 males and 478 females) aged over 60 years and 438 cases (208 males and 230 females) aged over 80 years. The clinical history of patients was obtained from the family and police examination records. For cases with a history of hospital visits, medical records were provided by the primary physician.

### Tissue sampling and pathological assessment

All brains were fixed in 20% buffered formalin for a minimum of 2 weeks before sampling. Specimens that are routinely sampled at our department have been described in a previous report [27] and are shown in Additional file 1: Figure S1. All sections were cut and stained with luxol fast blue-hematoxylin and eosin. Gallyas–Braak and Holzer staining was also performed. Routine immunohistochemistry was performed for samples of the frontal lobe, temporal lobes including the amygdala and hippocampus, basal ganglia, and midbrain of all cases to detect phosphorylated tau (clone AT8, 1:1000; Endogen, Woburn, MA), phosphorylated α-synuclein (clone LB508, 1:500; Zymed, San Francisco, CA), TAR DNA binding protein-43 (TDP-43; 1:5000; Protein Tech Group, Chicago, IL), glial fibrillary acidic protein (clone ZCG 29, 1:1000, Nichirei Tokyo, Japan), and β-amyloid (Aβ; clone 6F/3D, 1:50; Novocastra Vector Labs, Burlingame, CA). Antibody binding was detected using a biotin-streptavidin detection system (Nichilei, Tokyo, Japan) using 3,3′-diaminobenzidine as the chromogenic substrate. If positive findings were detected in the preliminary immunohistochemistry, an additional staining procedure was performed for subsequent sections. Staining for three- and four-repeat tau (Merck-Millipore, Billerica, MA) was also performed in cases positive for AT8. The pathological staging of neurofibrillary tangles (NFTs) was evaluated according to the modified Braak stages of NFT burden using AT8, Gallyas–Braak [28]. The density of neuritic plaques was evaluated in accordance with the Consortium to Establish a Registry for Alzheimer’s disease (CERAD) criteria using thioflavin-S and Aβ immunostaining [29]. The extent of senile plaques in the brain was evaluated using the criteria of Thal et al. [30]. Based on these results, the degree of AD-related neuropathological change was divided into four categories based on the National Institute on Aging-Alzheimer’s Association (NIA-AA) guidelines [31]: none, low, intermediate, and high. The pathology of Lewy body (LBs) disease was assessed according to the Third Consensus Guidelines for Dementia with LBs and the Braak stages for the development of Parkinson’s disease-related pathology using α-synuclein immunohistochemistry [32–34]. AGs were detected using Gallyas–Braak staining, and the pathological staging of AGs was assessed following the AGD system proposed by Saito et al. [11]. We used the National Institute of Neuronal Disorders and Stroke criteria to neuropathologically diagnose PSP [35, 36]. The pathological type of TDP-43 proteinopathy was assessed according to the stages of AD [37] and the classification system for frontotemporal lobar degeneration-TDP pathology [38].

### Investigation of predominant AGD subjects

Because AGD cases often have comorbid pathology, we extracted predominant AGD cases who had no comorbid pathology to examine the clinical significance of AGD. Predominant AGD cases were defined as cases with only mild AD pathology (Braak tau stage 2 or below, Thal phase 2 or below, or CERAD A or below) and without LBs or TDP-43 pathology. Controls were defined as cases with only mild AD lesions of the same severity as the above.

### Statistical analysis

Differences in continuous variables were analyzed using Student’s *t*-tests, and differences in ordinal variables were analyzed using chi-square tests. Spearman’s rank correlation test was used to analyze correlations. Although pathological stage was an ordinal variable, it was analyzed as a continuous variable. A propensity score was used to adjust for bias. Bonferroni correction was performed for multiple comparisons, and significance was set at *P* < 0.05. SPSS Statistics version 26 (IBM Corporation, Armonk, NY) and JMP 14.3 (JMP Statistical Discovery LLC, Cary, NC) were used for all analyses.

## Results

### Epidemiology

**Fig. 2:**
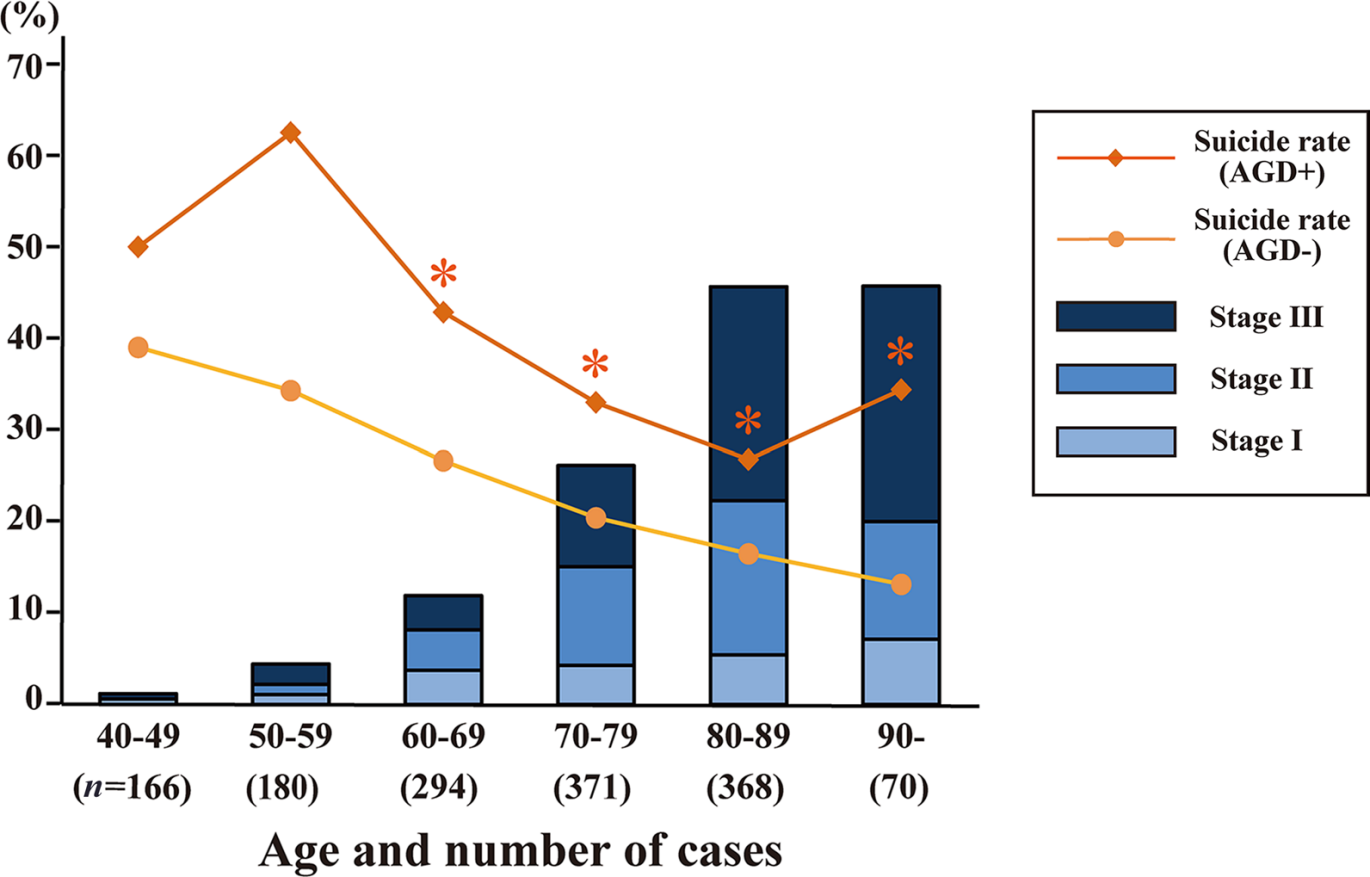
Frequency of argyrophilic grain disease (AGD) and suicide rate in each age group. The stage of AGD increased with age (Spearman’s rank correlation test *P* < 0.001). Suicide rates were significantly higher among AGD cases aged 60 years and above than non-AGD cases (*chi-square test *P* < 0.05)

**Fig. 3:**
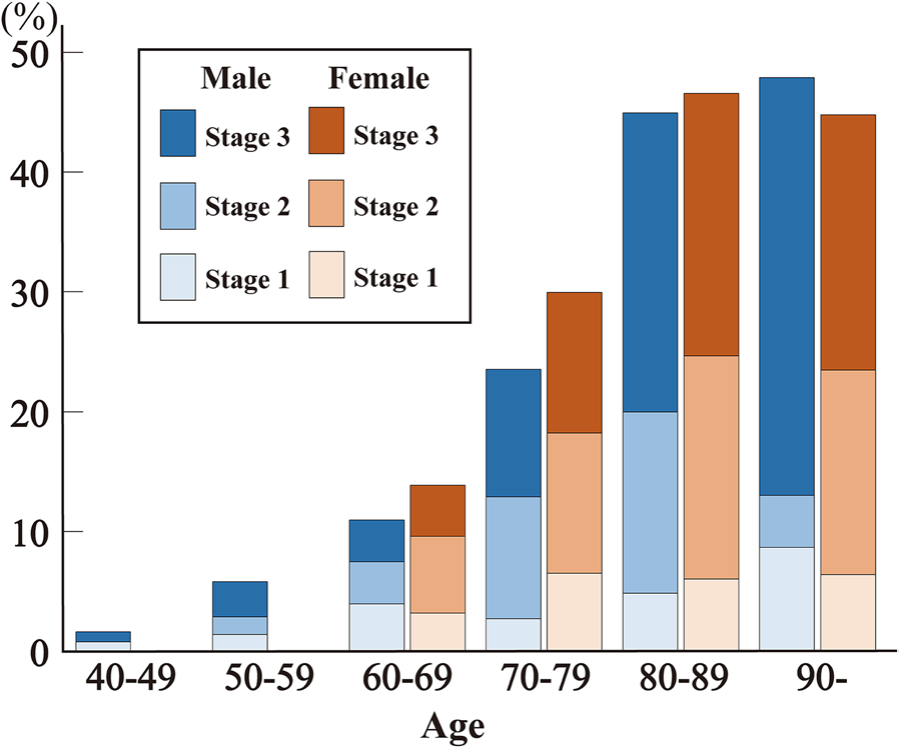
Frequency of argyrophilic grain disease (AGD) by age and sex. The frequency and stage of AGD did not differ significantly between men and women

Of the 1449 cases, we detected 342 AGD cases (23.6%; 177 males and 165 females), with a mean age of 79.7 years. The frequency of AGD was higher than that in numerous other studies (Table 1). The 1107 cases without AGD (704 males and 405 females) had a mean age of 67.0 years. There were significant differences in age and sex between the two groups (Table 2). The youngest AGD case was a 46-year-old male reported previously [26], and the oldest AGD case was 100 years old. The frequency and stage of AGD increased with age (Spearman’s rank correlation test *P* < 0.001), and 1.2% of patients aged 40–49 years and 45.7% of patients aged over 80 years had AGD. Pathological AGD stage also increased with age, and 23.7% of patients aged over 80 years were classified as AGD stage 3 (Fig. 2). Neither frequency nor AGD stage differed by sex in any age group (Fig. 3).

**Table 1:** Prevalence rates of argyrophilic grain disease (AGD) in previous studies and the current study

Study (year)	Number of cases (prevalence rate)	Mean age ± SD of AGD cases (in population)	Male/female ratio of AGD cases (in population)	Case selection method
Martinez-Lage and Munoz (1997) [41]	17/300 (5.6%)	77.0 ± 5.1 (61.3 ± 15.8)	8/9 (167/133)	Silver
Braak and Braak [5]	125/2536 (4.9%)	77.0 ± 8.8 (NA)	59/66 (NA)	Silver
Saito et al. (2004) [11]	449/1241 (36.2%)	NA (80.6 ± 8.9)	NA	Silver + immunohistochemistry
Togo et al. (2005) [19]	33/836 (8.5%)	83.7 ± 7.4 (79.9 ± NA)	15/18 (187/199)	Silver
Josephs et al. (2008) [39]	57/359 (15.9%)	NA	NA	Silver + immunohistochemistry
Rodriguez et al. (2016) [7]	152/983 (15.5%)	78.9 ± 9.4 (74.0 ± 11.7)	59/93 (473/510)	Immunohistochemistry
Current study (2023)	342/1449 (23.6%)	79.7 ± 9.0 (70.0 ± 14.1)	177/165 (881/568)	Silver

Study (year)	Under 60 years	60–69 years	70–79 years	Over 79 years
*Prevalence in each age group*				
Saito et al. (2004) [11]	0% (0/14)	17.6% (19/108)	31.2% (132/423)	42.8% (298/696)
Ferrer et al. (2008) [15]	10% (NA) *Under 61 years	17% (NA) *61–70 years	30% (NA) *71–80 years	43% (NA) *Over 80 years
Current study 2023)	2.9% (10/346)	11.9% (35/294)	26.1% (97/371)	45.7% (200/438)

*AGD* argyrophilic grain disease, *NA* not applicable, *SD* standard deviation

**Table 2:** Clinicopathological features of argyrophilic grain disease (AGD) and AGD-negative cases

	Positive cases (*n* = 342)	Negative cases (*n* = 1107)	Age- and sex-matched positive cases (*n* = 314)	Age- and sex-matched negative cases (*n* = 314)	Age-, sex-, and comorbid pathology-matched positive cases (*n* = 313)	Age-, sex- and comorbid pathology-matched negative cases (*n* = 313)
Age, mean ± SD, years	79.7 ± 9.0****	67.0 ± 14.1	78.8 ± 8.7	78.8 ± 8.7	79.0 ± 8.9	79.5 ± 9.0
Sex (male/female)	177/165^####^	704/403	170/144	166/148	168/145	169/144
BMI, mean ± SD	20.1 ± 3.6****	21.3 ± 4.5	20.3 ± 3.6	20.2 ± 3.9	20.2 ± 3.6	20.1 ± 3.7
Cause of death						
Suicide, *n* (%)	109 (31.9)	286 (25.8)	100 (31.9)^##^	64 (20.4)	103 (32.9)^####^	50 (20.2)
Homicide, *n* (%)	2 (0.6)	21 (1.9)	1 (0.3)	3 (1.0)	1 (0.3)	5 (1.6)
Accidental death, *n* (%)	175 (51.2)^#^	494 (44.6)	162 (51.6)	175 (55.7)	158 (50.5)	172 (55.0)
Natural causes, *n* (%)	52 (15.2)^####^	297 (26.8)	47 (15.0)^#^	71 (22.6)	48 (15.3)^#^	73 (23.3)
Past medical history						
Dementia, *n* (%)	80 (23.4)^####^	78 (7.1)	73 (23.3)^##^	42 (13.4)	68 (21.7)	49 (15.7)
Medical history of psychiatric disease, *n* (%)	51 (14.9)	161 (14.5)	50 (15.9)	37 (11.8)	49 (15.7)^#^	29 (9.3)
Pathological findings						
Brain weight, mean ± SD, g	1299.8 ± 145.6****	1360.4 ± 157.9	1305.6 ± 145.9	1300.3 ± 158.1	1304.8 ± 147.1	1309.8 ± 163.8
Heart weight, mean ± SD, g	362.1 ± 76.6**	380.4 ± 96.8	363.9 ± 76.3	375.2 ± 85.6	362.1 ± 74.4	370.1 ± 84.3
Braak AD tau stage, mean ± SD	3.5 ± 1.3****	2.1 ± 1.6	3.5 ± 1.3****	3.1 ± 1.5	3.4 ± 1.2	3.5 ± 1.3
Thal amyloid beta phase, mean ± SD	1.8 ± 1.6****	1.1 ± 1.4	1.7 ± 1.6	1.8 ± 1.5	1.8 ± 1.6	1.9 ± 1.6
CERAD amyloid beta stage, mean ± SD	1.3 ± 1.2****	0.8 ± 1.1	1.3 ± 1.2	1.4 ± 1.2	1.3 ± 1.2	1.4 ± 1.3
AD criteria in NIA-AA, High, *n* (%)	51 (14.9)^####^	77 (7.0)	49 (15.6)	41 (13.1)	42 (13.4)	59 (18.9)
Lewy pathology, *n* (%)	92 (26.9)^####^	144 (13.0)	84 (26.8)^#^	63 (20.1)	80 (25.6)	67 (21.4)
TDP-43 proteinopathy, *n* (%)	46 (13.5)^####^	25 (2.3)	41 (13.1)^##^	19 (6.1)	27 (8.6)	19 (6.1)
PSP pathology, *n* (%)	44 (12.9)^####^	31 (2.8)	38 (12.1)^##^	16 (5.1)	32 (10.2)	24 (7.7)

*AGD* argyrophilic grain disease; *AD* Alzheimer’s disease; *TDP-43* TAR DNA binding protein 43; *PSP* progressive supranuclear palsy; *SD* standard deviation; *CERAD* Consortium to Establish a Registry for Alzheimer’s disease; *NIA-AA* National Institute on Aging-Alzheimer’s Association; *BMI* body mass index. **P < 0.01; ****P < 0.0001 Student’s *t*-test; ^#^*P* < 0.05; ^##^ *P* < 0.01; ^####^*P* < 0.0001 chi-square test vs. negative cases

### Comorbid pathology

In the AGD cases, 51 (14.9%) had a high degree of AD pathology according to NIA-AA criteria, 92 (26.9%) had LB pathology, 46 (13.5%) had TDP-43 pathology, and 44 (12.9%) had PSP pathology. These conditions were more frequently observed in AGD patients than in AGD-negative cases. After matching for age and sex, the comorbidity of these conditions remained significantly higher in the AGD group than in the AGD-negative group, except for Aβ pathology. AD-related tau comorbidity was more severe in AGD cases than in non-AGD cases; however, the comorbidity rate of advanced-stage AD did not differ between the AGD and non-AGD groups (Table 2).

The severity and comorbidity of these pathologies tended to increase with the stage of AGD, which was not observed in the age-matched AGD-negative group (Table 3). In addition, there was a sex difference in comorbidity; TDP-43 was significantly more common in women than in men, and PSP lesions were significantly more common in men than in women (Additional file 1: Tables S1 and S2).

**Table 3:** Clinicopathological features of each argyrophilic grain disease (AGD) pathological stage

	Negative cases (*n* = 1107)	Negative cases aged over 70 years (*n* = 480)	Stage I (*n* = 55)	Stage II (*n* = 126)	Stage III (*n* = 161)
Age, mean ± SD, years	67.0 ± 14.1	80.3 ± 6.3	76.2 ± 10.0****	79.9 ± 8.1	80.8 ± 9.0
Sex (male/female)	704/403	262/218	28/27	60/66	89/72
BMI	21.3 ± 4.5	20.2 ± 3.9	20.7 ± 3.4	19.6 ± 3.8	20.2 ± 3.5
Cause of death					
Suicide, *n* (%)	286 (25.8)	83 (17.3)	19 (34.6)^##^	37 (29.4)^##^	53 (32.9)^####^
Homicide, *n* (%)	21 (1.9)	8 (1.7)	1 (1.8)	0 (0.0)	1 (0.6)
Accidental death, *n* (%)	494 (44.6)	280 (58.3)	25 (45.5)	65 (51.6)	85 (52.8)
Natural causes, *n* (%)	297 (26.8)	106 (22.1)	10 (18.2)	22 (17.5)	20 (12.4)^##^
Past medical history					
Dementia, *n* (%)	78 (7.1)	70 (14.6)	13 (23.6)	32 (25.4)^##^	35 (21.7)^#^
Medical history of psychiatric disorder, *n* (%)	161 (14.5)	37 (7.7)	8 (14.6)	17 (13.5)^#^	26 (16.2)^##^
Pathological findings					
Brain weight, mean ± SD, g	1360.4 ± 157.9	1299.4 ± 152.9	1306.2 ± 159.7	1297.1 ± 136.4	1299.7 ± 148.3
Heart weight, mean ± SD, g	380.4 ± 96.8	376.4 ± 86.7	360.8 ± 69.0	358.6 ± 76.6*	365.2 ± 79.3
Braak AD tau stage, mean ± SD	2.1 ± 1.6	3.1 ± 1.3	3.1 ± 1.2	3.5 ± 1.2*	3.7 ± 1.3****
Thal amyloid beta phase, mean ± SD	1.1 ± 1.4	1.9 ± 1.5	1.6 ± 1.6	1.9 ± 1.6	1.7 ± 1.7
CERAD amyloid beta stage, mean ± SD	0.8 ± 1.1	1.4 ± 1.2	1.2 ± 1.2	1.5 ± 1.2	1.3 ± 1.2
AD criteria in NIA-AA, High, *n* (%)	77 (7.0)	70 (14.6)	3 (5.5)	26 (20.6)	22 (13.7)
Lewy pathology, *n* (%)	144 (13.0)	100 (20.8)	7 (12.7)	37 (29.4)^#^	48 (29.8)^#^
TDP-43 pathology, *n* (%)	25 (2.3)	21 (4.4)	2 (3.6)	16 (12.7)^###^	28 (17.4)^####^
PSP pathology, *n* (%)	31 (2.8)	28 (5.8)	5 (9.1)	11 (8.7)	28 (17.4)^####^

*AGD* argyrophilic grain disease, *AD* Alzheimer’s disease, *PART* primary age-related tauopathy, *TDP-43* TAR DNA binding protein 43, *PSP* progressive supranuclear palsy, *SD* standard deviation, *CERAD* Consortium to Establish a Registry for Alzheimer’s disease, *NIA-AA* National Institute on Aging-Alzheimer’s Association, *BMI* body mass index. *P < 0.05; ****P < 0.0001 Student’s *t*-test, ^#^*P* < 0.05; ^##^*P* < 0.01; ^###^*P* < 0.001; ^####^*P* < 0.0001 chi-square test vs. negative cases

### Clinical appearance of AGD

In the AGD cases, 80 (23.4%) patients had dementia, although the difference was not significant when controlled for age, sex, and comorbid pathology. Fifty-one subjects (15.2%) had a history of psychiatric visits. The number of cases for each cause of death was 175 (51.2%) for accidental death, 109 (31.9%) for suicide, and 52 (15.2%) for natural causes. The rates of suicide and medical history of psychiatric disorders were significantly higher in these AGD cases than in the AGD-negative cases matched for age, sex, and comorbid pathology (Table 2).

The suicide rate of AGD cases was higher than that of AGD-negative cases in all age groups, although the difference was significant in only the 60 years and older age group. Moreover, the relative risk of suicide was 2.61 (1.01–6.73) in the 90 years and older group (Fig. 2). The suicide rates for each AGD stage were 34.9% for stage 1, 29.4% for stage 2, and 32.9% for stage 3. Rates did not differ between stages; however, they were significantly higher in the AGD cases than in the AGD-negative cases across all stages (Table 3).

The relative risk of suicide was 1.72 (1.30–2.26) and was statistically significant in female cases, with a relative risk of 1.80 (1.28–2.52), but not in male cases (Additional file 1: Tables S1 and S2). The relative risk of suicide increased to 2.27 (1.20–4.30) and 6.50 (1.58–26.76) in AGD cases with Lewy body pathology and PSP, respectively, and decreased to 0.88 (0.38–2.10) in AGD cases with advanced AD pathology. Dementia cases were significantly more common in the AGD group (23.4%) than in the non-AGD group. The relative risk of dementia was 1.38 (1.00–1.93) in patients with only AGD and increased to 1.73 (0.99–3.02) and 3.75 (1.93–7.29) in AGD cases with Lewy body pathology and severe AD pathology, respectively (Table 4).

**Table 4:** Relative suicide and dementia risk of cases with argyrophilic grain disease (AGD) and comorbid pathologies

Pathological comorbidity	Relative suicide risk	Relative dementia risk	Control group
AGD	1.72 (1.30–2.26)^####^	1.38 (1.00–1.93)	AGD(−)
AGD + AD (high)	0.88 (0.38–2.10)	3.75 (1.93–7.29)^####^	AGD(−) and AD (none, low, or intermediate)
AGD + Lewy	2.27 (1.20–4.30)^##^	1.73 (0.99–3.02)^#^	AGD(−) and Lewy(−)
AGD + TDP-43	2.20 (0.83–5.81)	1.0 (0.58–1.74)	AGD(−) and TDP-43(−)
AGD + PSP	6.50 (1.58–26.76)^##^	1.5 (0.60–3.78)	AGD(−) and PSP(−)

*AGD* argyrophilic grain disease; *AD* Alzheimer’s disease; *Lewy* Lewy body pathology, *TDP-43* TAR DNA binding protein 43; *PSP* progressive supranuclear palsy, ^#^*P* < 0.05; ^*##*^*P* < 0.01; ^####^*P* < 0.0001 chi-square vs. negative cases

### Predominant AGD cases

Thirty-nine cases (11.4%) were predominant AGD cases, of whom 16 (41.0%) were suicidal and four (10.3%) had dementia. A comparison of cases between these AGD cases and those with the same level of mild AD pathology only (i.e., the control group), matched for age, sex, and AD pathology, showed a significantly higher suicide rate and a significantly lower rate of natural death in the predominant AGD group (Table 5).

**Table 5:** Clinicopathological features of predominant argyrophilic grain disease (AGD) cases

	Predominant AGD cases (*n* = 39)	Control cases (*n* = 589)	Age-, sex-, and comorbid pathology-matched predominant AGD cases (*n* = 37)	Age-, sex-, and comorbid pathology-matched control cases (*n* = 37)
Age, mean ± SD, years	72.5 ± 12.1****	59.3 ± 12.0	71.7 ± 2.0	70.9 ± 2.0
Sex (male/female)	23/16	417/172	22/15	23/14
BMI, mean ± SD	21.0 ± 3.8	21.9 ± 4.7	21.0 ± 3.9	21.5 ± 4.7
Cause of death				
Suicide, *n* (%)	16 (41.0)	183 (31.1)	15 (40.5)^#^	7 (18.9)
Homicide, *n* (%)	0 (0.0)	11 (1.9)	0 (0.0)	0 (0.0)
Accidental death, *n* (%)	18 (46.2)	216 (36.7)	17 (46.0)	16 (43.2)
Natural causes, *n* (%)	5 (12.8)^#^	173 (29.4)	5 (13.5)^#^	14 (37.8)
Past medical history				
Dementia, *n* (%)	4 (10.3)^####^	2 (0.3)	3 (8.1)	0 (0.0)
Medical history of psychiatric disease, *n* (%)	7 (18.0)	110 (18.7)	7 (18.9)	5 (13.5)
Pathological findings				
Brain weight, mean ± SD, g	1332.5 ± 151.4*	1395.9 ± 148.0	1338.3 ± 152.5	1377.9 ± 119.1
Heart weight, mean ± SD, g	368.5 ± 66.7	384.8 ± 100.5	370.2 ± 67.4	397.8 ± 85.2
Braak AD tau stage, mean ± SD	1.8 ± 0.5****	1.0 ± 0.8	1.8 ± 0.5	1.8 ± 0.4
Thal amyloid beta phase, mean ± SD	0.4 ± 0.7	0.3 ± 0.6	0.4 ± 0.7	0.3 ± 0.6
CERAD amyloid beta stage, mean ± SD	0.2 ± 0.4	0.2 ± 0.4	0.2 ± 0.4	0.2 ± 0.4

*AGD* argyrophilic grain disease; *AD* Alzheimer’s disease; *SD* standard deviation; *CERAD* Consortium to Establish a Registry for Alzheimer’s disease; *BMI* body mass index. *P < 0.05; ****P < 0.0001 Student’s *t*-test, ^#^*P* < 0.05; ^####^*P* < 0.0001 chi-square test vs. negative cases

## Discussion

Although we identified AGD cases using Gallyas–Braak staining, which is considered less sensitive than immunohistochemistry, the frequency of AGD was higher than that in most other studies. With a few exceptions, most forensic autopsies in Japan are performed under the criminal code and are performed when the cause of death is suspected to be unnatural or linked to a crime [27]. Although our previous study of a large sample of forensic autopsy cases may not represent the general Japanese population because of a lower frequency of bedridden cases in the terminal phase of various diseases, we showed that the frequency of PSP and CBD may be higher than expected in a forensic autopsy sample [27, 40]. In PSP patients, in particular, a high incidence of lethal traumatic injury and suicide may be associated with the high frequency of PSP in our previous study. Therefore, a high frequency of unnatural death cases, especially suicide cases, may contribute to the high frequency of AGD in the present study. In contrast, an autopsy study conducted in Japan by Saito et al. showed a high frequency of AGD (36.2%) in hospital autopsy cases, despite the exclusion of suicide cases [11]. Although the mean age of participants in the study by Saito et al. (80.6 ± 8.9 years) was higher than that of the participants in the present study [11], the frequency of AGD in each age group was similar across both studies. The difference in the frequency of AGD may be associated with the difference in the age of the sampled cases. It is worth noting that the frequency of neurodegenerative diseases as investigated by autopsy may vary depending on the investigated population.

Although studies have shown that AGD is associated with a higher rate of mild cognitive impairment [18, 41], several studies have shown a high frequency of AGD pathology in control cases [7, 16, 17]. Rodriguez et al. revealed that in AGD cases without other neuropathological findings (8 of 152 AGD cases), half of them had some degree of cognitive decline, whereas 59% of their 152 AGD subjects were cognitively normal [7]. In addition, Davis et al. identified AGD in 23% of 59 cognitively normal older adult subjects [16], and Knopman et al. identified 12 cases of AGD among 59 cognitively normal older adult subjects [17]. Wurms et al. showed varied clinical manifestations in younger AGD subjects (aged under 75 years) without significant comorbid neuropathological lesions [42]. In our study, we used a propensity score to compare AGD subjects with age-, sex-, and comorbid pathology-matched control cases and showed that AGD was not a distinct neuropathological lesion of dementia. This result is consistent with the conclusion of several previous studies [5, 7, 19, 37, 41]. However, we cannot conclude from our findings that AGD is not entirely associated with the development of dementia because the neurological findings based on clinical evaluations in many of our forensic autopsy cases tended to be less comprehensive than previous studies targeting hospitalized patients or brain donation program participants. In summary, we assumed that the effect of AGD on dementia may be relatively weak and that other pathologies, such as AD or LB pathology, may be more strongly associated with dementia in subjects with AGD.

We showed that AGD may be a distinct contributing factor to psychiatric disorders in the older adult population. Several psychiatric conditions, such as aggression, irritability, depression, psychosis, and mild dementia, have been shown to be associated with AGD [19, 21, 43, 44], and several studies have revealed that these psychological changes tend to precede the appearance of dementia in patients with AGD, whereas dementia tends to appear before psychological changes in patients with AD [6, 19, 21]. Of these various psychiatric symptoms, late-life depression (LLD) has been shown to cause cognitive dysfunction and increase the risk of dementia and AD by two folds; however, the nature of the relationship between LLD and dementia has not yet been explored [45]. Togo et al. [19] also showed that amnesia and emotional disorders are frequently present in patients with AGD, whereas other cognitive functions tend to be spared relative to the severity of amnesia. Shioya et al. investigated the pathological appearance of 11 older adult cases with bipolar disorder and showed that AGD may be associated with late-onset bipolar disorder in middle-aged or older subjects [46]. Furthermore, Jellinger [3] showed that personality changes and frontal lobe signs are much more prominent in AGD patients than in dementia patients. Nagao et al. [44] showed that late-onset delusions occur significantly more frequently in patients with AGD than in those with minimal AD pathology alone and suggested that AGD is associated with the occurrence of late-onset schizophrenia and delusional disorders.

Although the specific mechanism underlying psychiatric symptoms in AGD patients has not yet been established, psychiatric disorders tend to occur in various conditions after the limbic region and temporal cortex become affected [47–51]. Investigations in subjects with Parkinson’s disease (PD) have shown that dysfunctions of the limbic system and brainstem nuclei are associated with depression [51]. Moreover, other selective and morphological limbic dysfunctions, such as amnesia and behavioral changes, have been reported to be caused by cerebrovascular disease, traumatic brain injury, epilepsy [47, 48], herpes simplex encephalitis, and paraneoplastic syndrome [49, 52]. Specifically, the amygdala, which is a crucial hub of the emotional processing neural system, has been implicated in LLD pathophysiology [50]. In addition to the initial and predominant distribution of AGs in patients with AGD, we found that subjects in the early pathological stage of AGD are also at risk of developing a psychiatric disorder. This suggests that the involvement of the amygdala is an important mechanism in the progression of psychiatric disorders in subjects with AGD. Numerous functional magnetic resonance imaging (fMRI) studies have also shown the involvement of the amygdala in various psychiatric disorders, including depression [53]. However, morphometric analyses of the amygdala of patients with depression using clinical imaging data have provided inconsistent results, including an increase, a decrease, and no change [54]. Surdhar et al. [55] reported that amygdala volumes are significantly smaller in PD patients with mild depressive symptoms than in healthy controls. Leal et al. investigated older adults with and without depressive symptoms using fMRI to discern signals in the hippocampal subfields and amygdala nuclei and showed that the disruption of the amygdala–entorhinal–hippocampal network is associated with LLD [56]. Davey et al. suggested that the amygdala is involved in the generation of negative affect that characterizes depression [57]. These radiological findings suggest an association between AGD and late-onset psychiatric disorders, such as LLD, due to the morphological alteration of the amygdala caused by the accumulation of AGs.

A key result of the current study is that AGD is a distinct risk factor for suicide in the older adult population. As an individual ages, quality of life may decrease because of a decline in physical and cognitive abilities or illnesses. Chronic diseases and reduced strength lead to a sense of worthlessness and anxiety, which can eventually result in depression [58–60]. Epidemiological studies have identified various risk factors for suicidal thoughts and behaviors; however, whether these findings can generalize to other cultures remains unclear [58]. Obuobi-Donker et al. revealed that adults aged 60 years and above are at a risk of developing LLD, which can expose them to suicidal behaviors. Psychiatric and physical illnesses, functional impairments, and social/economic factors may also contribute to suicidal behavior in older adults [59]. A study of 538 suicide cases indicated that suicidal behavior is a product of the interaction of numerous factors; however, no single independent risk factor was found [60]. In the present study, suicide rates did not differ between AGD stages, but the suicide rate was significantly higher in AGD cases, even at stage-1 AGD, than in non-AGD cases. Therefore, the involvement of the amygdala and the accumulation of AGs may be associated with suicidal behavior. Detailed neuropathological examinations of suicide victims investigating the correlation between suicide and neurodegenerative diseases are scarce. To date, three studies have investigated AD pathology in victims of suicide, although conclusions varied. Rubio et al. [22] examined the autopsies of 28 individuals who had died of suicide and found that the frequency of AD pathology was higher than that in the control group. In contrast, Peisah et al. [23] did not find a positive correlation between suicide and AD pathology. Moreover, Matschke et al. found no difference in the frequency of AD-type pathology in the brain between suicide victims and control cases [24]. However, these studies were all limited by the lack of standard neuropathological investigations and evaluations of AD pathology. Furthermore, the amygdala was not studied. We showed that impaired function of the limbic system, including the amygdala, may be a neuropathological substrate for both psychiatric disorders and suicidal behavior. However, further clinicopathological investigations are required to explore why there was no significant difference in the rate of suicide between different stages of AGD.

We found a significantly higher frequency of suicide in women in our sample, although men also showed a similar tendency. Several epidemiological studies have focused on sex difference in risk factors for LLD and/or suicide [59–62]. Although older adult women are more likely to experience depression than older adult men [61], men are more likely to die by suicide, whereas women are more likely to attempt suicide [59]. The mechanism underlying sex difference in suicidal behavior remains unclear because only a few studies have been conducted, and such studies have not found a significant interaction between various risk factors and suicidal behavior [62]. It is uncertain why the suicide rate was significantly higher in the AGD group than in the non-AGD group in women only, especially because the frequency of AGD did not differ between men and women. Possible causes are the difference in the sensitivity of the limbic system to AGs between men and women or the presence of other potent accelerating factors for suicidal behavior in men. Crestani et al. investigated 538 suicide cases and showed that chronic and debilitating diseases, often accompanied by profound psychological symptoms, are powerful stimuli to prompt suicide among men, whereas mental state is a significant risk factor for women, as the majority of them had depression [60].

In older adult subjects, mixed pathology (i.e., mixed neurodegenerative pathology or mixed neurodegenerative and other pathologies) is thought to lower the threshold for developing cognitive impairment and dementia; moreover, the severity of mixed pathologies increases with age and correlates with the severity of clinical symptoms [63]. We revealed that the overlap of AGD with PSP or LB pathology may increase the risk of suicide in the present study. Indeed, we previously reported that AGD may be a significant risk factor for suicide attempts in older adults with a clinical history of acute post-stroke depression or incipient PSP lesions [27, 64]. Neuropsychiatric changes are common in PSP patients, especially apathy, and depression is less common [65], although another study suggested that depression is highly common in patients with PSP [66]. Although we did not examine the detailed neuropsychiatric appearance of subjects with overlapping AGD and PSP or LB pathology, the overlap of AGD with other pathologies may increase the amount of pathological substrate in the limbic system, which may lead to the development of psychiatric disorders, including suicide attempts. On the other hand, although it remains unclear why the overlapping AD-related tau pathology decreased the risk of suicide in the present study, we speculate that the ability to attempt suicide and/or complete suicide is diminished as cognitive impairment progresses owing to the deterioration of AD pathology. A recent clinical study reported that subjects younger than 65 years in the earlier stages of dementia had a 6.69-times (95% CI 1.49–30.12) higher suicide risk compared with subject without dementia [67]. Further investigations of neuropsychiatric symptoms, including those in the earlier stages of dementia, and their association with suicidal behavior in elderly subjects with autopsy-proven neurodegenerative disease are essential.

In addition to a certain level of bias in our study population, our study was also limited by the lack of clinical information of some cases, which was mainly because they lacked severe clinical symptoms or had low rates of neurologist consultation. In particular, the level of education, the standard of living and other possible confounding factors such as debilitating illness, overall weakness, and the financial situation of each patient were not evaluated. In addition, we identified AGD subjects by examining the left hemisphere of the brain. However, neuropathological asymmetry in AGD subjects as revealed by Adachi et al. has been shown to be associated with the progression of psychiatric symptoms, including suicide and/or dementia [68]. Finally, we did not examine whether a larger amount of AGs are associated with the development of clinical symptoms. Thus, additional clinicopathological investigations, especially more detailed clinical information and investigation of the association between suicide and other abnormal protein accumulations, may be required.

## Conclusion

Although the frequency of AGD in autopsy studies varies depending on the population studied, we show here that the frequency of AGD in a Japanese forensic autopsy series is 25.2% in individuals aged over 40 years. The frequency increases with age, where 1.2% of patients in their 40s have AGD, and 45.7% of patients aged over 80 years have AGD. AGD is identified as a significant and distinct risk factor for psychiatric hospital visits and completion of suicide but not for dementia. Overlapping PSP and LB pathology increases the risk of suicide, whereas overlapping advanced AD pathology decreases the risk of completing suicide. Morphological changes in the limbic system, including an increase in AGs in the amygdala, may be associated with the development of psychiatric disorders and suicide attempts in older adults with AGD. The development of methods to detect AGs in clinical practice may help prevent suicide in older adults.

## Supplementary Information

**Additional file 1: Fig. S1.** Low power view of the histological specimen (luxol fast blue-hematoxylin and eosin). **Table S1.** Clinicopathological features of male argyrophilic grain disease cases. **Table S2.** Clinicopathological features of female argyrophilic grain disease cases.

## Data Availability

The datasets analyzed during the current study are available from the corresponding author on reasonable request.

## Abbreviations

Aβ: β-Amyloid
AD: Alzheimer’s disease
AGs: Argyrophilic grains
AGD: Argyrophilic grain disease
CBD: Corticobasal degeneration
CERAD: Consortium to Establish a Registry for Alzheimer’s disease criteria
fMRI: Functional magnetic resonance imaging
LBs: Lewy bodies
LLD: Late-life depression
NFTs: Neurofibrillary tangles
NIA-AA: National Institute on Aging-Alzheimer’s Association guidelines
PD: Parkinson’s disease
PSP: Progressive supranuclear palsy
TDP-43: TAR DNA binding protein-43
