# Supplementary material for: Argyrophilic grain disease is common in older adults and may be a risk factor for suicide: a study of Japanese forensic autopsy cases
Source: Transl Neurodegener. 2023 Apr 1;12:16. doi: 10.1186/s40035-023-00352-2 (PMC10067165; doi:10.1186/s40035-023-00352-2)
Supplement: Supplementary file 1 — Additional file 1: Fig. S1. Low power view of the histological specimen (luxol fast blue-hematoxylin and eosin). Table S1. Clinicopathological features of male argyrophilic grain disease cases. Table S2. Clinicopathological features of female argyrophilic grain disease cases. [file 40035_2023_352_MOESM1_ESM.pdf]

Additional file 1

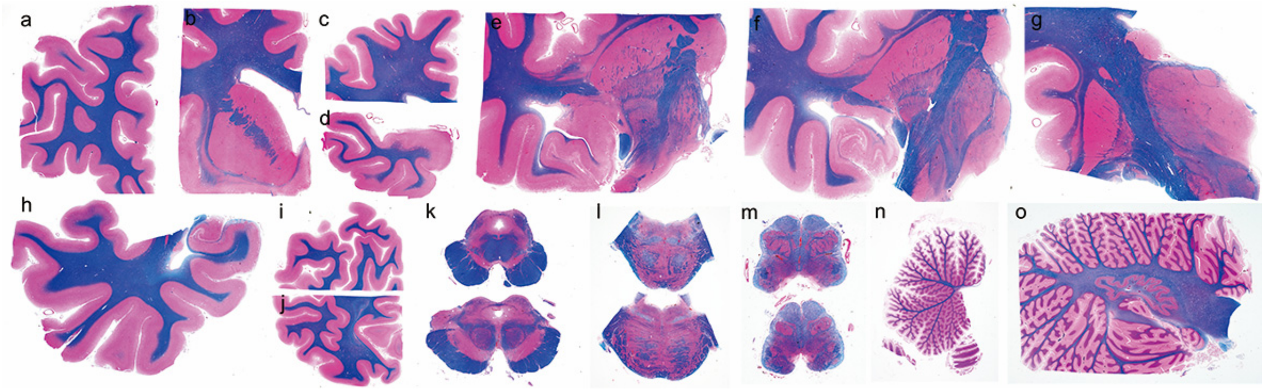

Figure S1 Low power view of the histological specimen (luxol fast blue-hematoxylin and eosin). **a** Frontal lobe. **b** Nucleus accumbens. **c** Motor cortex. **d** Anterior temporal lobe. **e** Basal ganglia and amygdala. **f** Anterior hippocampus and temporal lobe. **g** Thalamus and subthalamic nucleus. **h** Posterior hippocampus and temporal lobe. **I and j** Occipital lobe. **k** Midbrain. **l** Pons. **m** Medulla oblongata. **n** Cerebellar vermis. **o** Dentate nucleus

Table S1. Clinicopathological features of male argyrophilic grain disease cases

	Positive cases (n = 177)	Negative cases (n = 704)	Age-matched positive cases (n = 166)	Age-matched negative cases (n = 166)	Age- and comorbid pathology-matched positive cases (n = 156)	Age- and comorbid pathology-matched negative cases (n = 156)
Age, mean ± SD, years	77.9 ± 9.5****	65.1 ± 13.6	77.3 ± 9.4	77.3 ± 9.4	77.4 ± 9.6	77.4 ± 9.9
BMI	20.3 ± 3.5***	21.7 ± 4.4	20.4 ± 3.3	20.6 ± 4.0	20.2 ± 3.5	20.6 ± 3.6
Cause of death						
Suicide, n (%)	38 (21.5)	142 (20.2)	38 (22.9)#	22 (13.3)	35 (22.4)	22 (14.1)
Homicide, n (%)	0 (0.0)	13 (1.9)	0 (0.0)	2 (1.2)	0 (0.0)	1 (0.6)
Accidental death, n (%)	104 (58.8)$^{\#\#}$	332 (47.2)	96 (57.8)	111 (66.9)	92 (59.0)	95 (60.9)
Natural causes, n (%)	33 (18.6)$^{\#\#}$	213 (30.3)	30 (18.1)	30 (18.1)	28 (18.0)	38 (24.4)
Past medical history						
Dementia, n (%)	39 (22.0)$^{\#\#\#\#}$	35 (5.0)	38 (22.9)$^{\#\#\#}$	15 (9.0)	32 (20.5)	22 (14.1)
Medical history of psychiatric disease, n (%)	16 (9.0)	72 (10.2)	15 (9.0)	11 (6.6)	16 (10.3)	11 (7.1)
Pathological findings						
Brain weight, mean ± SD, g	1366.9 ± 140.7****	1418.5 ± 143.9	1370.6 ± 141.4	1368.0 ± 157.2	1362.8 ± 143.0	1368.7 ± 161.6
Heart weight, mean ± SD, g	389.0 ± 81.9*	406.0 ± 97.7	389.3 ± 81.6	398.2 ± 95.2	382.1 ± 79.9	397.9 ± 83.2
Braak AD tau stage, mean ± SD	3.5 ± 1.2****	1.8 ± 1.5	3.5 ± 1.2****	2.7 ± 1.5	3.3 ± 1.1	3.3 ± 1.3

Thal amyloid beta phase, mean ± SD	1.6 ± 1.6****	0.9 ± 1.3	1.6 ± 1.6	1.6 ± 1.6	1.6 ± 1.6	1.5 ± 1.6
CERAD amyloid beta stage, mean ± SD	1.2 ± 1.2****	0.7 ± 1.0	1.2 ± 1.2	1.3 ± 1.2	1.2 ± 1.2	1.2 ± 1.3
AD criteria in NIA-AA, High, n (%)	24 (13.6)####	36 (5.1)	24 (14.5)	18 (10.8)	20 (12.8)	27 (17.3)
Lewy pathology, n (%)	42 (23.7)####	83 (11.8)	38 (22.9)	30 (18.1)	33 (21.2)	29 (18.6)
TDP-43 pathology, n (%)	18 (10.2)####	16 (2.3)	18 (10.8)	11 (6.6)	12 (7.7)	13 (8.3)
PSP pathology, n (%)	25 (14.1)####	16 (2.3)	23 (13.9)##	9 (5.4)	16 (10.3)	11 (7.1)

AGD argyrophilic grain disease; *AD* Alzheimer's disease; *TDP-43* TAR DNA binding protein 43; *PSP* progressive supranuclear palsy; *SD* standard deviation; *CERAD* Consortium to Establish a Registry for Alzheimer's disease; *NIA-AA* National Institute on Aging-Alzheimer's Association; *BMI* body mass index. *$P < 0.05$, ***$P < 0.001$, ****$P < 0.001$ Student's t-test; #$P < 0.05$, ## $P < 0.01$, ###$P < 0.001$, ####$P < 0.0001$ chi-square test vs negative cases

Table S2. Clinicopathological features of female argyrophilic grain disease cases

	Positive cases (*n* = 165)	Negative cases (*n* = 403)	Age-matched positive cases (*n* = 142)	Age-matched negative cases (*n* = 142)	Age- and comorbid pathology-matched positive cases (*n* = 136)	Age- and comorbid pathology-matched negative cases (*n* = 136)
Age, mean ± SD, years	81.7 ± 7.9****	70.5 ± 14.3	80.3 ± 7.6	80.3 ± 7.6	80.2 ± 7.6	80.4 ± 8.5
BMI	19.8 ± 3.8*	20.5 ± 4.5	20.1 ± 3.7*	19.2 ± 3.6	19.9 ± 3.9	19.7 ± 3.8
Cause of death						
Suicide, *n* (%)	71 (43.0)	144 (35.7)	66 (46.5)$^{\#\#\#\#}$	39 (27.5)	63 (46.3)$^{\#\#\#}$	35 (25.7)
Homicide, *n* (%)	2 (1.2)	8 (2.0)	1 (0.7)	3 (2.1)	2 (1.5)	3 (2.2)
Accidental death, *n* (%)	71 (43.0)	162 (40.2)	58 (40.9)	65 (45.8)	54 (39.7)	65 (47.8)
Natural cause, *n* (%)	19 (11.5)$^{\#\#}$	84 (20.8)	15 (10.6)$^{\#\#}$	33 (23.2)	15 (11.0)$^{\#\#}$	31 (22.8)
Past medical history						
Dementia, n (%)	41 (24.9)$^{\#\#\#\#}$	43 (10.7)	34 (23.9)	27 (19.0)	30 (22.1)	27 (19.9)
Medical history of psychiatric disease, n (%)	35 (21.2)	89 (22.1)	34 (23.9)	19 (13.4)	32 (23.5)$^{\#}$	17 (12.5)
Pathological findings						
Brain weight, mean ± SD, g	1227.8 ± 112.9**	1258.7 ± 126.9	1231.6 ± 116.9	1220.1 ± 114.5	1237.2 ± 113.9	1219.4 ± 125.8
Heart weight, mean ± SD, g	333.1 ± 58.1	335.5 ± 76.9	336.5 ± 58.1	342.1 ± 73.5	336.3 ± 59.1	347.9 ± 79.9
Braak AD tau stage, mean ± SD	3.5 ± 1.4****	2.5 ± 1.6	3.4 ± 1.3	3.5 ± 1.4	3.3 ± 1.3	3.5 ± 1.3

Thal amyloid beta phase, mean ± SD	2.0 ± 1.6****	1.4 ± 1.6	1.9 ± 1.6	2.1 ± 1.6	1.9 ± 1.6	2.1 ± 1.6
CERAD amyloid beta stage, mean ± SD	1.5 ± 1.2****	1.0 ± 1.2	1.4 ± 1.2	1.6 ± 1.2	1.4 ± 1.2	1.6 ± 1.2
AD criteria in NIA-AA, High, n (%)	27 (16.4)$^{\#}$	41 (10.2)	20 (14.1)	30 (21.1)	19 (14.0)	26 (19.1)
Lewy pathology, n (%)	50 (30.3)$^{\#\#\#\#}$	61 (15.1)	41 (28.9)	35 (24.7)	36 (26.5)	36 (26.5)
TDP-43 pathology, n (%)	28 (17.0)$^{\#\#\#\#}$	9 (2.2)	19 (13.4)$^{\#\#}$	6 (4.2)	36 (26.5)	41 (30.2)
PSP pathology, n (%)	19 (11.5)$^{\#\#\#\#}$	15 (3.7)	13 (9.1)	9 (6.3)	9 (6.6)	7 (5.2)

AGD argyrophilic grain disease; *AD* Alzheimer's disease; *TDP-43* TAR DNA binding protein 43; *PSP* progressive supranuclear palsy; *SD* standard deviation; *CERAD* Consortium to Establish a Registry for Alzheimer's disease; *NIA-AA* National Institute on Aging-Alzheimer's Association; *BMI* body mass index. *$P < 0.05$, ****$P < 0.001$ Student's t-test; $^{\#}P < 0.05$, $^{\#\#}P < 0.01$, $^{\#\#\#}P < 0.001$, $^{\#\#\#\#}P < 0.0001$ chi-square test vs negative cases
